# Identifying the region responsible for *Brucella abortus* MucR higher-order oligomer formation and examining its role in gene regulation

**DOI:** 10.1038/s41598-018-35432-1

**Published:** 2018-11-22

**Authors:** Luciano Pirone, Joshua Edison Pitzer, Gianluca D’Abrosca, Roberto Fattorusso, Gaetano Malgieri, Emilia Maria Pedone, Paolo Vincenzo Pedone, Roy Martin Roop, Ilaria Baglivo

**Affiliations:** 1 Department of Environmental, Biological and Pharmaceutical Sciences and Technologies, University of Campania “Luigi Vanvitelli”, Caserta, 81100 Italy; 2 Institute of Biostructures and Bioimaging, C.N.R, Naples, 80134 Italy; 3 Department of Microbiology and Immunology, Brody School of Medicine, East Carolina University, Greenville, NC USA

## Abstract

MucR is a member of the Ros/MucR family of prokaryotic zinc-finger proteins found in the α-proteobacteria which regulate the expression of genes required for the successful pathogenic and symbiotic interactions of these bacteria with the eukaryotic hosts. The structure and function of their distinctive zinc-finger domain has been well-studied, but only recently the quaternary structure of the full length proteins was investigated demonstrating their ability to form higher-order oligomers. The aim of this study was to identify the region of MucR involved in higher-order oligomer formation by analysing deletion and point mutants of this protein by Light Scattering, and to determine the role that MucR oligomerization plays in the regulatory function of this protein. Here we demonstrate that a conserved hydrophobic region at the N-terminus of MucR is responsible for higher-order oligomer formation and that MucR oligomerization is essential for its regulatory function in *Brucella*. All these features of MucR are shared by the histone-like nucleoid structuring protein, (H-NS), leading us to propose that the prokaryotic zinc-finger proteins in the MucR/Ros family control gene expression employing a mechanism similar to that used by the H-NS proteins, rather than working as classical transcriptional regulators.

## Introduction

The Ros/MucR protein family^1,2^ includes prokaryotic zinc-finger proteins such as Ros from *Agrobacterium tumefaciens*^3^ and MucR from *Brucella* spp.^4–6^, both of which regulate genes required for the virulence of these strains in their respective plant and animal hosts^2,5–7^. Also included in this family are MucR from *Sinorhizobium meliloti*^7,8^, and from *Sinorhizobium fredii*^9^; RosR from *Rhizobium etli*^10^ and from *Rhizobium leguminosarum*^11^ which regulate genes required for the successful symbiosis of these bacteria with plants. Additionally, the structural homologs MucR1 and MucR2 play important roles in coordinating the orderly expression of the cell cycle genes in *Caulobacter crescentus*^12^. Many structural features related to the DNA-binding domains of Ros from *Agrobacerium tumefaciens* and Mls from *Mesorhizobium loti* have been described^13–21^. One of the interesting features of these and other Ros/MucR homologs is that direct binding studies suggest that these proteins recognize A-T rich regions in and around bacterial promoters that have little sequence consensus^12,22–25^. Recently, we demonstrated that the AT-rich DNA targets sites for the *Mesorhizobium* Mls and *Brucella* MucR contain T-A steps, and that these proteins contact DNA mostly in the minor groove and are able to form higher-order oligomers^26^. Furthermore, we have shown that MucR from *Brucella abortus* is able to recognize multiple AT-rich sites in the promoter of its own gene and that it is a heat-stable protein with a Tm of 63 °C^27^. The ability to bind AT-rich sites containing T-A steps in the minor groove, the capacity to oligomerize and the heat-stability are also features of another prokaryotic protein family, the histone-like nucleoid structuring proteins, H-NS^28–34^. H-NS proteins not only play important roles in nucleoid compaction, but they also serve as gene silencers, preventing the potentially toxic expression of bacterial genes acquired by horizontal gene transfer^35–37^ and repressing the gratuitous expression of virulence genes in bacterial pathogens^38,39^. One of the important features of H-NS proteins with regard to their ability to serve as gene silencers is their capacity to recognize AT-rich DNA-target sites containing T-A steps in and around promoters. They use these sequences as nucleation sites to form higher-order oligomers that prevent RNA polymerase access to these promoters^40,41^.

Here, we identify a hydrophobic region as responsible for the higher-order oligomer formation at the N-terminus of the prokaryotic zinc-finger protein MucR and definitively demonstrate the importance of MucR oligomerization for its regulatory function in *Brucella*. Based on the results presented here, together with previously published findings^26,27^, we propose that the prokaryotic zinc-finger proteins in the Ros/MucR family control gene expression by employing a mechanism similar to that used by the H-NS proteins, rather than working as classical transcriptional regulators.

## Results

### A conserved hydrophobic region located at the N-terminus of MucR is responsible for higher-order oligomer formation

**Figure 1:**
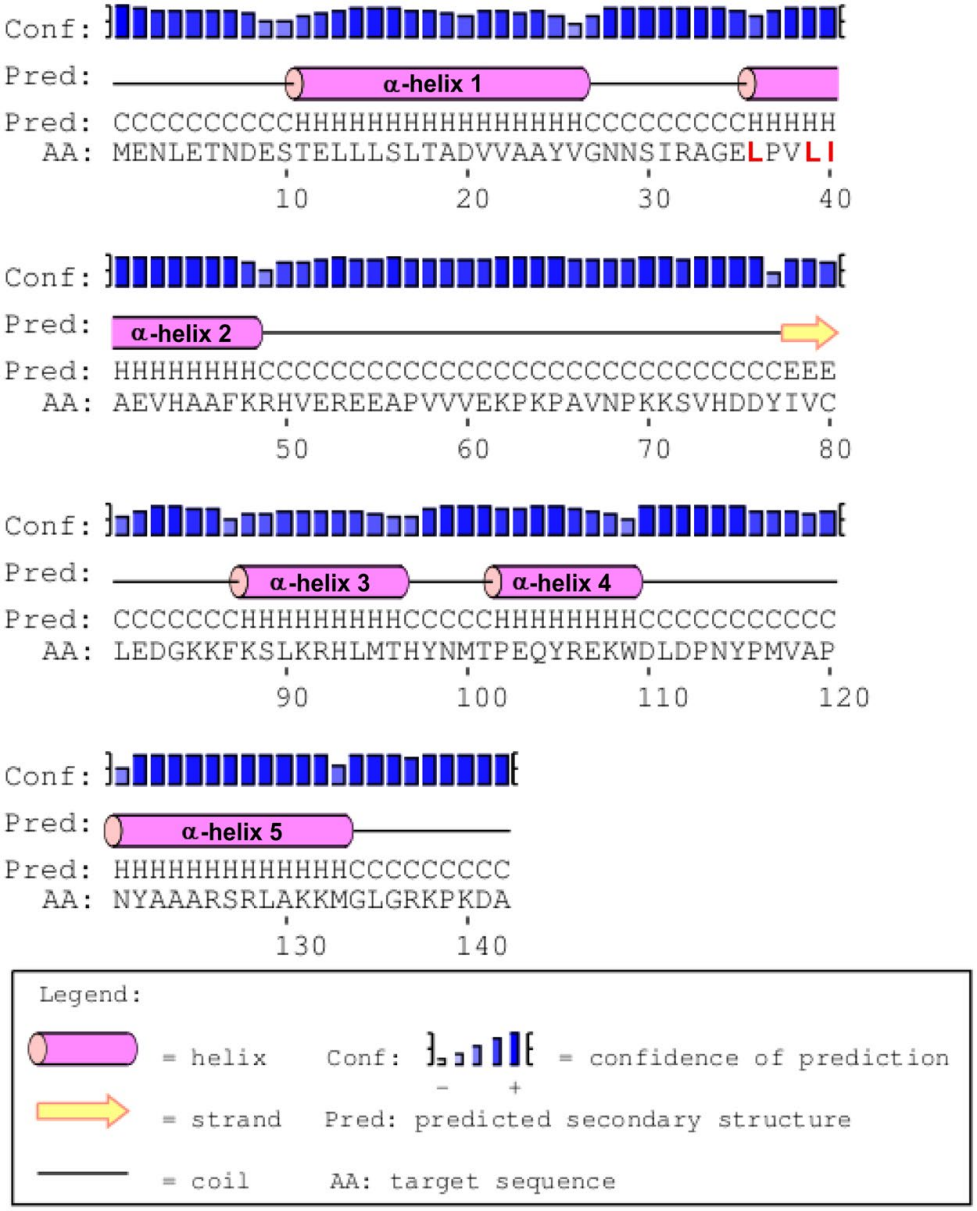
Secondary structure prediction of MucR by PSIPRED tool. The predicted α-helices are indicated and numbered in the barrels; the amino acids mutated in the MucR^L36L39I40A^ are reported in red.

**Figure 2:**
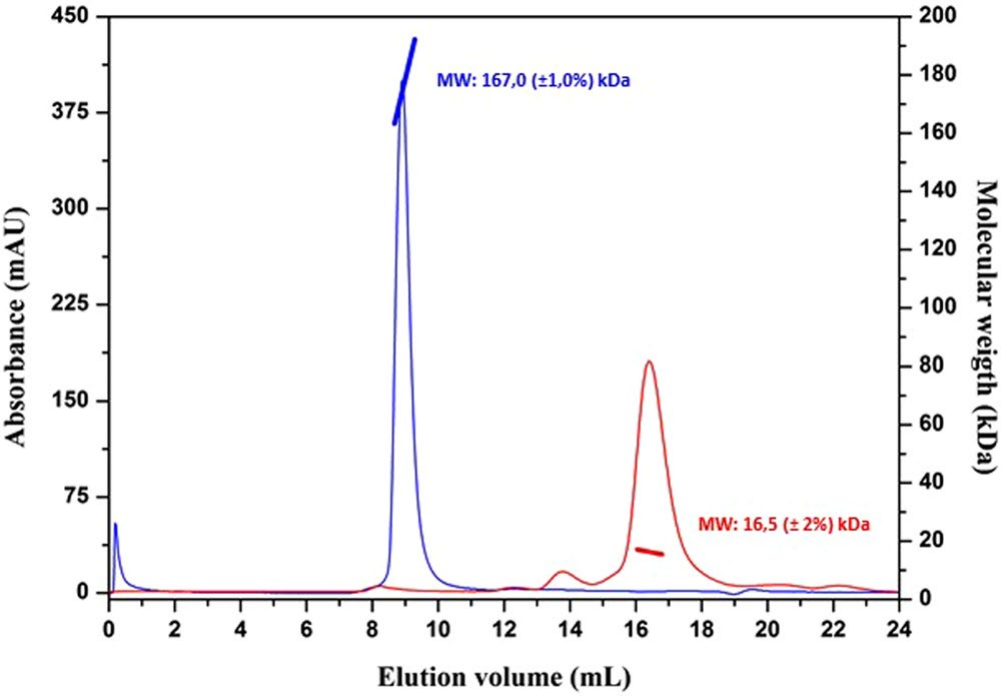
Gel-filtration light-scattering analysis of MucR (blue) and MucR^L36L39I40A^ (red). The solid lines represent the signals of the eluted proteins at 280 nm. The molecular weight of each peak calculated by LS of MucR (reported in blue) and MucR^L36L39I40A^ (reported in red) is also reported.

As previously reported^26^, the N-terminal region of MucR and of the homologous Ml proteins is responsible for higher-order oligomer formation. Using the PSIPRED tool^42^, we obtained a secondary structure prediction of the *Brucella* MucR (Fig. 1). In the N-terminal region, two α-helices were predicted, one extending from the threonine in position 11 to the valine in position 27 and the other from the leucine in position 36 to the lysine in position 48. In an attempt to identify the amino acids in the N-terminal region of MucR involved in oligomerization, we designed the deletion mutant MucR_33–142_, in which the first 32 amino acids including the first putative α-helix, were deleted, and the deletion mutant MucR_45–142_, in which the deletion was extended to the amino acid in position 44 breaking down the second predicted α-helix (Fig. 1). To investigate the oligomeric state of MucR_33–142_ and MucR_45–142_, we performed a static Light Scattering (LS) analysis (Table 1). The results obtained under the conditions tested show that the deletion mutant MucR_33–142_ is still able to form higher-order oligomers showing a decameric state, whereas the deletion mutant MucR_45–142_ turns out to be in a monomeric state. The same result was obtained with the deletion mutant MucR_57–142_ (Table 1) lacking the N-terminal region and comprising only the region corresponding to the Ros DNA-binding domain whose structure was solved by NMR^15^. These results identify the region spanning from the alanine in position 33 to that in position 44 as responsible for oligomerization. This amino acid sequence contains two leucines in position 36 and 39, and an isoleucine in position 40 that constitute a hydrophobic region highly conserved in MucR homologs (Suppl. Fig. 1). We thus designed a mutant version of MucR_33–142_, named MucR_33-142_mut, in which these three conserved amino acids were mutated into alanines. Analysed by LS, MucR_33-142_mut turned out to be a monomer under the conditions tested, indicating that the conserved residues leucine 36, leucine 39 and isoleucine 40 are involved in higher-order oligomer formation (Table 1). To investigate the role of this highly conserved hydrophobic region in the full-length MucR, we expressed and purified the MucR^L36L39I40A^ mutant, a version of MucR in which the leucines 36 and 39, and the isoleucine 40 are mutated into alanines. Performing LS analysis, we found that MucR^L36L39I40A^ turns out to be a monomer losing its quaternary decameric structure (Fig. 2) indicating that the here identified highly conserved hydrophobic region in the second putative α-helix at the N-terminus of MucR is responsible for higher-order oligomer formation.

**Table 1:** Light Scattering (LS) analysis of the MucR deletion mutants.

Protein	Theoretical molecular weight monomer	Experimental Molecular weight by Static LS	ExMw/ThMw
MucR	16024 Da	167000 (±1, 0%) Da	10.40
MucR_33–142_	12559 Da	132600 (±2, 0%)	10.50
MucR_45–142_	11330 Da	11510 (±0, 6%)	1.01
MucR_57–142_	9905 Da	10740 (±3%)	1.08
MucR_33–142 mut_	12334 Da	12690 (±0, 7%)	1.02

The theoretical molecular weight of the monomer calculated by ProtParam tool (http://web.expasy.org/protparam/) is reported in the second column; the results by LS in the third column; the ratio between the experimental weights found and the monomer weights are reported in the fourth column.

### NMR analysis of MucR structure and oligomerization

**Figure 3:**
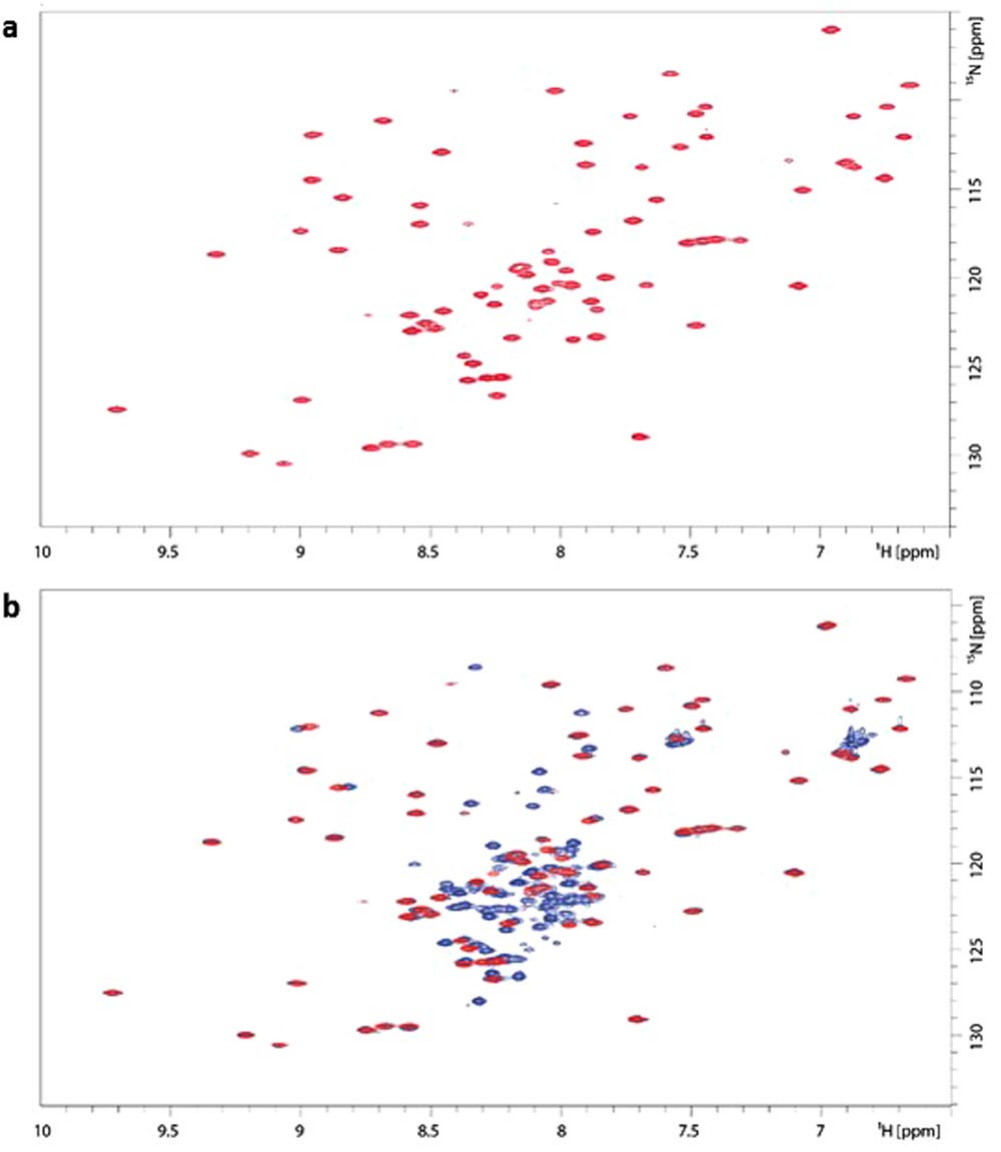
(**a**) ^1^H-^15^N HSQC spectrum of MucR_57–142_ acquired at 600 MHz and at 298 K; (**b**) superposition of MucR_57–142_ and MucR^L36L39I40A 1^H^−15^N HSQC spectra acquired at 600 MHz and at 298 K. The spectrum of MucR_57–142_ is in red whereas that of MucR^L36L39I40A^ is in blue.

We performed a structural analysis of the studied proteins by means of NMR spectroscopy. The NMR study started with the analysis of the DOSY experiment recorded for the MucR_57–142_ deletion mutant. This experiment gave a diffusion coefficient (D_t_) of 1.22 (±0.12) *10^−10^ m^2^ sec^−1^, which is very similar to the value measured for Ros87, thus confirming the monomeric form of MucR_57-142_ also at the NMR concentration used^43^. Figure 3, panel a, reports the ^1^H-^15^N HSQC spectrum of the same protein. The spectrum is consistent with a well-defined native structure with extensive tertiary interactions. It shows a large number of resonances well dispersed over a chemical shift range of about 3 ppm in the proton dimension and 25 ppm in the ^15^N dimension. This feature clearly indicates the presence of β-strands, as this kind of secondary structure is typically associated with a good dispersion of the NMR signals. The more crowded region in the centre of the spectrum is likely to contain resonances from helical structures which lead to a minor degree of dispersion.

The same spectra were then recorded for the wild-type full-length protein MucR and for the full-length mutant MucR^L36L39I40A^. Consistent with the data acquired using other techniques, the wild-type full-length protein gave very low quality spectra bearing only a few broad signals suggesting the presence of high-order oligomers. The behaviour of the protein remained the same also when lowering the concentration, indicating a concentration independent tendency of this protein to form oligomers. Quite opposite is the behaviour of MucR^L36L39I40A^. This triple point mutant gave good quality spectra (Fig. 3b) which are consistent with a well-defined monomeric tertiary structure (D_t_ = 1.18 (±0.13) * 10^−10^ m^2^ sec^−1^). Figure 3, panel b, reports the superposition of the spectra recorded for the MucR_57–142_ mutant with those of MucR^L36L39I40A^ and shows how the DNA-binding domain, apart from minor local differences, is essentially contained within the structure of MucR^L36L39I40A^.

### MucR^L36L39I40A^ fails to form the slow mobility protein-DNA complex observed in EMSA of MucR

**Figure 4:**
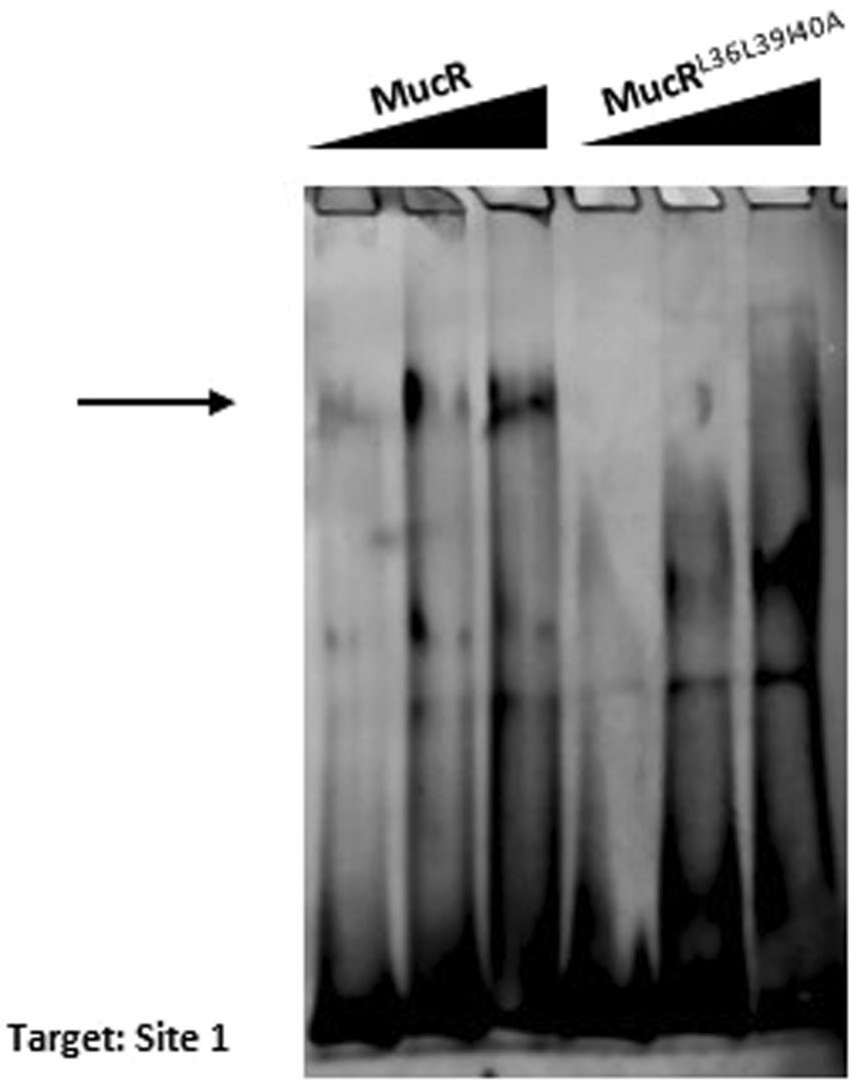
MucR and MucR^L36L39I40A^ binding to Site 1 by EMSA. The increasing amount of the protein used is indicated on the top of the lanes as well as the name of the proteins. The target sequence Site1 was previously identified in the promoter of *mucR* gene at −174 bp from the ATG start codon^27^. The arrow indicate the protein/DNA complex formed by MucR with Site1 which does not appear in the EMSAs of MucR^L36L39I40A^.

To investigate the ability of the mutant protein MucR^L36L39I40A^ to bind DNA, we performed electrophoretic mobility shift assays (EMSAs) using one of the MucR DNA-binding site, named Site1, previously identified in the *mucR* gene promoter and located at −174 bp from the ATG start codon^27^. Comparing the protein/DNA complexes formed by MucR and MucR^L36L39I40A^, it is evident that the mutant protein is unable to form the slow mobility MucR/DNA complex indicated by the arrow in Fig. 4 and in Suppl. Fig. 2. It is likely that the mutant MucR^L36L39I40A^ cannot form higher-order oligomer bound to DNA, but only the DNA-protein complexes with lower molecular masses which turn to be faster in their electrophoretic mobility. The results observed by EMSAs are in line with NMR data, which show that the DNA-binding domain is not altered by the mutation at the N-terminus of the protein and with those obtained by LS which demonstrates that MucR^L36L39I40A^ is not able to form higher-order oligomers.

### The region of MucR responsible for oligomerization is necessary for its wild-type regulatory function in *B. abortus* 2308

**Figure 5:**
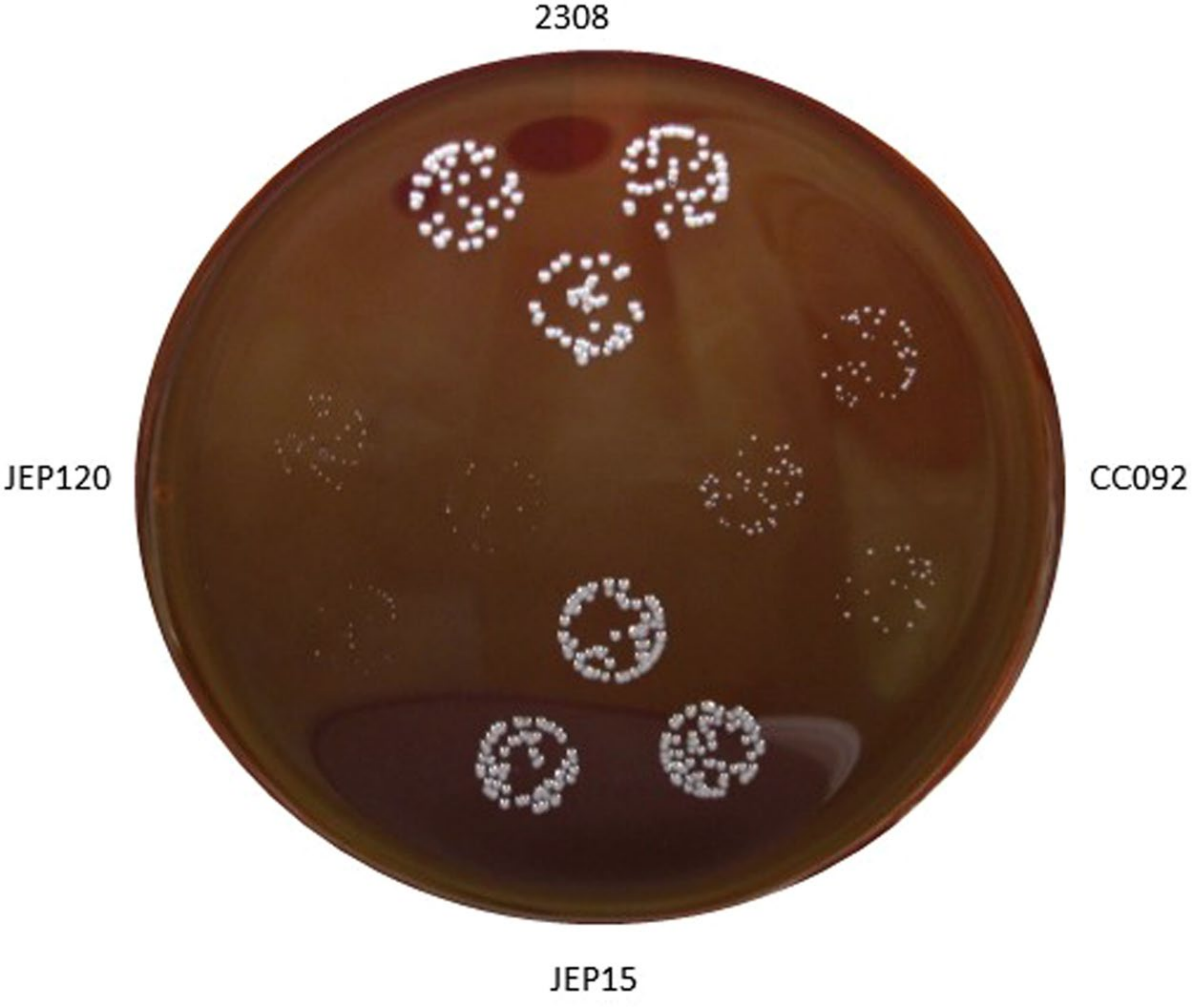
Colony formation by *B. abortus* 2308, an isogenic *mucR* mutant CC092 and derivatives of CC092 carrying plasmid-borne copies of *mucR* (JEP15) or *mucR*^L36L39I40A^ (JEP120) following 72 h incubation at 37 °C on Schaedler agar supplemented with 5% defibrinated bovine blood.

The *B. abortus mucR* mutant CC092 displays a delay in its ability to form colonies on agar plates compared to its parent strain *B. abortus* 2308^4^, and rescue of this growth defect by genetic complementation with plasmid-borne copies of mutated *mucR* alleles has proven to be a useful tool for structure/function analysis of the MucR protein. To investigate whether or not MucR requires the region responsible for oligomerization to rescue the growth defect exhibited by *B. abortus* CC092, we performed a genetic complementation experiment transforming this *mucR* mutant with the plasmids containing the wild-type gene *mucR* (pJep011) and the mutated *mucR*^*L36L39I40A*^ gene (pJep120). Transcription of the *mucR* wild-type and *mucR*^*L36L39I40A*^ in the *B. abortus* transformants was verified by q-RT PCR, and colony formation by these strains was monitored during cultivation on Schaedler blood agar supplemented with bovine blood^4^. Unlike the native *mucR* gene, a plasmid-borne copy of the *mucR*^*L36L39I40A*^ allele did not restore the capacity of the *B. abortus* CC092 *mucR* mutant to form the same sized colonies as the parental 2308 strain following 72 h growth on agar plates (Fig. 5). These results strongly suggest that oligomerization is required for the normal physiologic function of MucR.

**Figure 6:**
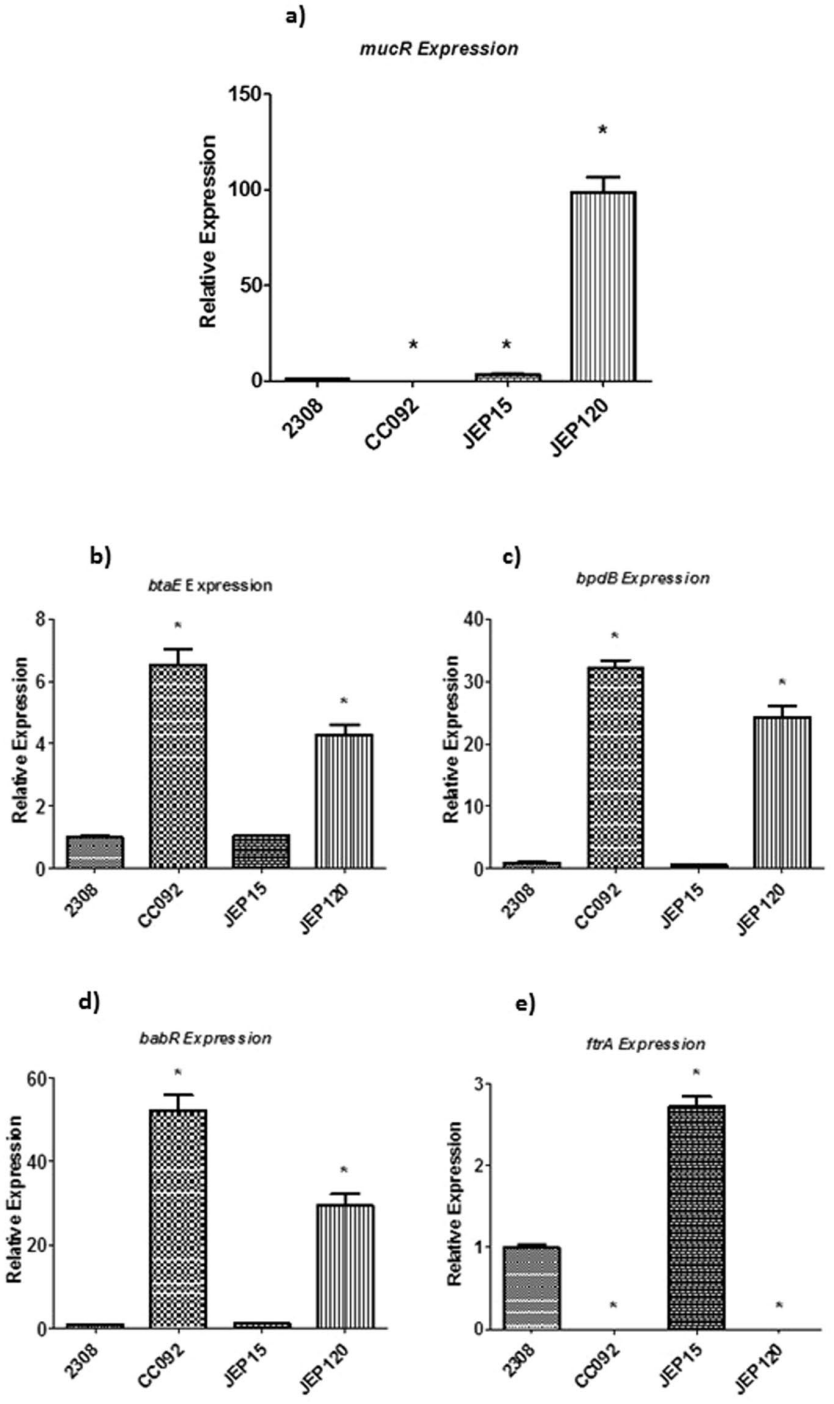
Relative expression of (**a**) *mucR*, (**b**) *btaE*, (**c**) *bpdB*, (**d**) *babR and* (**e**) *ftrA* in *B. abortus* 2308, an isogenic *mucR* mutant (CC092), the *mucR* mutant carrying a plasmid-borne copy of *mucR* (JEP15), and the *mucR* mutant carrying a plasmid-borne copy of the *mucR*^L36L39I40A^ (JEP120) determined by q-RT PCR. RNA was obtained from mid-log phase bacterial cultures grown in brucella broth. The results presented are from a single representative experiment with three technical replicates for each experimental condition. The experiment was repeated three times with equivalent results. *P < 0.05 for comparisons of 2308 vs. the other three strains using the Student t-test.

Microarray analysis indicates that MucR controls the expression of more than 90 genes in *B. abortus* 2308^4^. Genes that have been found to be direct targets of MucR repression by EMSA analysis (J. Pitzer, unpublished data) include the gene encoding the LuxR-type quorum sensing regulator BabR^44^, the gene encoding the c-diGMP degrading phosphodiesterase BpdB^45^, the gene encoding the polar adhesin BtaE^46^ and the *mucR* gene itself^4^. EMSA analysis (J. Pitzer, unpublished data) also indicates that the operon encoding the Fe^2+^ transporter FtrABCD^47^ is a direct target of MucR activation in *Brucella*. To investigate whether or not the MucR^L36L39I40A^ mutant is able to regulate these genes, we used q-RT PCR analysis to compare the transcription patterns of *babR*, *bpdB*, *btaE*, *mucR* and *ftrA* in *B. abortus* 2308, the isogenic *mucR* mutant CC092, and derivatives of the *mucR* mutant carrying plasmid-borne copies of the native *mucR* gene (JEP15) or the mutant *mucR*^*L36L39I40A*^ gene (JEP120). The results clearly show that the mutant MucR^L36L39I40A^ has lost its ability to repress *btaE*, *bpdB*, *babR* and *mucR* expression, or activate *ftrA* expression, with the same efficiency as the wild-type MucR (Fig. 6). These experimental findings strongly suggest that MucR oligomerization is essential for its regulatory function in *Brucella*.

## Discussion

The Ros/MucR prokaryotic zinc-finger proteins are global transcriptional regulators in the α-proteobacteria, where they work in concert with other transcriptional regulators to coordinate the expression of genes required for the symbiotic^8–10,48,49^ and pathogenic^3–6,50^ interactions of these bacteria with their eukaryotic hosts, and the orderly progression of the aquatic bacterium *Caulobacter crescentus* through its well-described developmental cycle^12^. Studies employing transposon mutagenesis^51^, transcriptomic^4,6,8,9,52^ and proteomic analyses^53^, and chromatin immunoprecipitation coupled with high throughput DNA sequencing (ChIPseq)^12^ have shown that many genes involved in basic metabolic and physiologic processes and virulence and symbiotic properties in a variety of different α-proteobacteria are regulated by Ros/MucR homologs, and in most cases these genes appear to be targets of Ros/MucR repression. The zinc-finger domains of these proteins have been studied in detail both structurally and functionally^1,13–20,54^. Previous studies have shown that these proteins bind AT-rich DNA sequences^12,13,22–25^. We recently demonstrated that AT-rich DNA sequences containing T-A steps are necessary and sufficient for DNA-binding of the Ros/MucR proteins and that these proteins contact DNA mainly in the minor groove^26^.

From a structural standpoint, the prokaryotic and eukaryotic C_2_H_2_ zinc-finger domains share similarities, including their ability to bind DNA, the tetrahedral coordination of a structural zinc ion and the presence of ββα secondary structures^15^. On the other hand, the prokaryotic C_2_H_2_ zinc-finger shows some peculiarities such as the second α-helix, the flexibility of the zinc-coordination sphere and the larger hydrophobic core that together with the zinc-finger motif constitutes the DNA-binding domain^1,14–18^. Our recent studies with full-length Ros/MucR proteins has determined that these proteins form higher-order oligomers^26^, are stable at high temperature and are able to bind multiple sites present in the promoters of their target genes^26,27^. These combined experimental findings have led us to propose that the Ros/MucR proteins regulate gene expression in the same manner as the DNA structuring protein H-NS, which is found in many other Gram-negative bacteria^55^.

In the present study, we define a hydrophobic region including residues L36, L39and I40 as being responsible for higher-order oligomer formation by the *Brucella* MucR, and show that a targeted mutation of this region is sufficient to get the protein to switch from a decameric state to a monomeric one. This finding together with the predicted structure of the MucR N-terminus suggests the possibility that the second α-helix comprising the hydrophobic region identified here at the N-terminus of MucR could be involved in the formation of higher-order oligomers. Our data clearly demonstrate that the absence of the first α-helix at the N-terminus of MucR is not sufficient to change the oligomeric state of the protein. In fact, the deletion mutant 33–142 containing this first α-helix is still able to form higher-order oligomers, whereas the 45–142 deletion mutant of MucR lacking the first α-helix and the hydrophobic region L36L39I40, presents only a monomeric state. The MucR^L36L39I40A^ mutant demonstrates that this hydrophobic region included in the second α-helix at the N-terminus of MucR has an essential role in oligomerization. More structural studies are necessary to understand how the interaction between MucR monomers might occur and whether or not other secondary structure elements have a role in stabilizing the higher-order oligomers. In H-NS more than one region is involved in higher-order oligomer formation^56–58^ whereas in the case of MucR, the mutation of three residues at the N-terminus is sufficient to get a switch from higher-order oligomers to a monomer. The presence of H-NS lower-order oligomers in solution was thoroughly investigated^29,57–63^ showing many different degrees of H-NS oligomers depending on both different experimental conditions and/or portions of the protein analysed. All the studies agreed in pointing out the crucial role of higher-order oligomer formation for proper H-NS function. Our findings with MucR cannot exclude the existence of lower-order oligomers which could be present at very low concentration in solution and contribute to the formation of higher-order oligomers similar to the H-NS. However, we can state that the main region forming higher-order oligomers is located at the N-terminus of MucR and that the hydrophobic region that this protein shares with all of the MucR/Ros homologs plays a major role in building the higher-order oligomers. Our preliminary results indicate that there are about 5.5 × 10^4^ MucR molecules for *B. abortus* cells, or about 3.6% of the total cellular protein (J. Pitzer, unpublished data). This is consistent with the number of H-NS and other nucleoid-associated proteins that have been reported in other bacteria which ranges from 10^4^ to 10^5^ molecules per cell corresponding to μM concentration^56,64^. This high intracellular level of MucR also indicates that the *in vitro* experimental conditions used here to observe its oligomerization are likely to be physiologically relevant.

The observation that the *mucR*^*L36L39I40A*^ allele cannot repress the *btaE*, *bpdB*, *babR* or *mucR* expression and cannot activate the *ftrA* expression in *B. abortus* indicates that MucR must be able to form oligomers to retain its wild-type regulatory function. These experimental findings also provide further support for our hypothesis that MucR plays a similar role to that proposed for H-NS in terms of its ability to serve as a transcriptional regulator. Its capacity to form extensive oligomers allows H-NS to compact DNA, and accordingly, it plays an important role in establishing and maintaining nucleoid structure in bacteria^59–66^. But the members of H-NS protein family also play an important role in regulating prokaryotic gene expression. The capacity of these proteins to bind to AT-rich regions in and around bacterial promoters, oligomerize and make these promoters inaccessible to RNA polymerase allows H-NS proteins to serve as global gene ‘silencers’^38,39,41^. Experimental evidence suggests that the gene silencing capacity of H-NS proteins is important for both protecting bacteria from the uncontrolled expression of xenogeneic genes acquired by horizontal gene transfer^30,32–39,56^ and preventing the gratuitous expression of genes that only provide bacteria with a fitness benefit in a particular environment (e.g. genes required for virulence in a mammalian host)^30–39^. Notably, previous genetic and biochemical studies suggest that MucR and its homologs function predominately as transcriptional repressors in the α-proteobacteria^4,6,8,12,51^ and that they work in concert with antagonistic transcriptional activators in these bacteria to ensure that virulence, symbiosis and cell cycle genes are only expressed when they are needed by these bacteria^67^. However, it is important to note that the inability of the *mucR*^*L36L39I40A*^ allele to activate *ftrA* transcription in *B. abortus* gene is also compatible with the proposed role of MucR as an H-NS-like regulator. Specifically, in addition to its negative effects, the nucleoid structuring role of H-NS proteins can also have positive effects on the transcription of some genes because this structuring provides easier access of RNA polymerase and transcriptional activators to their promoter regions^33^.

Previous studies have provided evidence that Ros/MucR proteins in other α-proteobacteria form oligomeric complexes^11,68^, and the data presented here suggest that the ability to form these complexes plays an important role in the capacity of these proteins to function as transcriptional regulators. The capacity to bind to low consensus AT-rich regions of DNA in and around the promoters of the genes they regulate also seems to be a shared feature of the Ros/MucR proteins that have been characterized^7,12,13,22–25,69^. Further biochemical and genetic studies will be necessary to fully understand how MucR homologs function as transcriptional regulators, but a model in which they recognize AT-rich sequences containing T-A steps in DNA as nucleation sites, bind other AT-rich regions, form high-order oligomers and prevent access to bacterial promoters by RNA polymerase is certainly consistent with the data presented here and with the information available in the literature with regard to the capacity of these proteins to serve as transcription repressors. This suggests that from a functional standpoint, perhaps the Ros/MucR family of zinc-finger prokaryotic proteins should be considered to be new members of the family of so-called H-NS-‘like’ gene silencers^30,32,35–37^. Although the role of H-NS as a global gene silencer is well established, not all bacteria, including the majority of the α-proteobacteria, possess an H-NS homolog^55^. However, other small nucleoid binding proteins that have no significant amino acid identity with H-NS have been found to perform a similar regulatory role. The MvaT and MvaU proteins in *Pseudomonas*^29,32,34^ and the Lsr2 protein in *Mycobacterium*^30^, for instance, are small nucleoid binding proteins that also recognize AT-rich regions containing T-A steps in DNA, bind to these regions and form oligomers, and serve as global repressors of gene expression.

## Conclusion

The experimental findings presented here show that the hydrophobic region defined by the amino acids L36L39I40 at the N-terminus of the *Brucella* MucR is required for this protein to be able to form higher-order oligomers and to perform its normal function as a transcriptional regulator. Based on these and earlier findings^26,27^, a new functional model is arising for the prokaryotic zinc-finger proteins in the Ros/MucR family. Specifically, these proteins appear to be binding to low consensus AT-rich regions in DNA and functioning as H-NS-‘like’ gene silencers, unlike their counterparts in eukaryotes which function mainly as DNA sequence-specific transcriptional regulators^70^.

## Methods

### Cloning, protein expression and purification

The DNA fragments encoding for MucR^L36L39I40A^, MucR_33–142_, MucR_33–142_mut, MucR_45–142_, MucR_57–142_ were generated by PCR. Primers were designed on the basis of the wild-type *mucR* gene sequence (GenBank: SHO30402.1) to get the deletions or the point mutations of the wild-type gene. The sequence of the primers are shown in the Suppl. Table 1; the template used for PCRs was the pET-22b(+) plasmid containing the wild-type *mucR* gene previously published^26^. The PCR products encoding for MucR_33–142_, MucR_33–142_mut, MucR_45–142_, MucR_57–142_ were digested with NdeI/EcoRI restriction enzymes and cloned into pET-22b(+) expression vector digested with the same enzymes; the PCR product encoding for MucR^L36L39I40A^ was digested with NcoI/EcoRI restriction enzymes and cloned into the pET-11D expression vector digested with NcoI/EcoRI as well. All the proteins were expressed as previously reported^1^ in *E. coli* host strain BL21(DE3) grown in Luria Bertani medium (for proteins to be analysed by Light Scattering) or grown in a minimal medium^1^ containing 0.5 g/L ^15^NH_4_Cl as the only nitrogen source (for proteins to be analysed by NMR). The expression was induced for 1 h at 28 °C with 1 mM IPTG. Protein purification was carried out as previously reported^1^. The proteins were eluted from a Mono S HR 5/5 cation exchange chromatography column in the 0.3–0.6 M NaCl concentration range.

### Light Scattering

For molecular weight measurements, a MiniDAWN Treos spectrometer (Wyatt Instrument Technology Corp.) equipped with a laser operating at 658 nm was used connected on-line to a size-exclusion chromatography column. Samples at 1 mg/ml were loaded onto a Superdex 200 column (10 × 30 cm, GEHealthcare) equilibrated in the same buffer used for the final purification procedure and connected to a triple-angle light scattering detector equipped with a QELS (Quasi-Elastic Light Scattering) module^71^. A constant flow rate of 0.5 ml/min was applied. Elution profiles were detected by a Shodex interferometric refractometer and a mini Dawn TREOS light scattering system. Data were analyzed using the Astra 5.3.4.14 software (Wyatt Technology). The experiments were carried out in duplicate.

### NMR spectroscopy

The NMR spectra of the proteins purified as described above were recorded at 298 K on a Bruker Avance III HD 600 MHz equipped with cryoprobe at the Department of Environmental, Biological and Pharmaceutical Science and Technology, University of Campania - Luigi Vanvitelli (Caserta, Italy). The NMR samples of MucR, MucR^L36L39I40A^ and MucR_57–142_ contained 200 μM or 500 μM of purified ^15^N labelled proteins in a 20 mM phosphate buffer, 0.3 M NaCl at pH = 6.8.

The ^1^H-^15^N HSQC spectra of MucR, MucR_57–142_ and MucR^L36L39I40A^ were acquired at 298 K using 256 complex points for ^15^N (F1) and 2048 for ^1^H (F2). The translational diffusion coefficient (Dtrans)^72^ was obtained by using the PFG diffusion measurements with the PG-SLED (pulse gradient-stimulated echo longitudinal encode-decode) sequence^73^.

Data were processed using the TopSpin 3.5 software (Bruker) and analyzed with the CARA software as previously reported^16^ (downloaded from cara.nmr.ch+).

### Electrophoretic Mobility Shift Assay (EMSA)

The EMSA experiments were performed as previously described^74,75^. In detail, 0.4, 0.6 or 0.8 μg of each protein were incubated 10 min on ice in binding buffer (25 mM HEPES pH 7.9, 50 mM KCl, 6.25 mM MgCl2, 5% glycerol) with 5 pmol of double-stranded oligonucleotides Site 1(5′-GTTGCCTATTATTAATGTAATATGGTTTGA-3′) previously published as a target site of MucR and located at −174 bp from the ATG start codon of *mucR* gene^27^. The protein/DNA ratio for each sample was 5, 8, 10 when 0.4, 0.6, 0.8 μg of protein were used respectively. To obtain a negative control, 0.8 μg of each protein were incubated with 5 pmol of the double-stranded oligonucleotide NC (5′-CGCGGCACGACCGCAGCGGTCGGGTGGCAC-3′) in the same binding buffer whose composition has been already described above. The total volume of each reaction mixture was 20 μl. After incubation on ice, the samples were loaded onto a 5% polyacrylamide gel in 0.5X TBE and run at room temperature for 70 min at 200 V. Gels were stained 20 minutes using Diamond™ Nucleic Acid Dye (Promega) following the manufacturer’s instructions and imaged by Typhoon Trio+ scanner (GE Healthcare). The results by EMSAs shown in this study are representative of more than 10 replicates.

### Genetic complementation of a Brucella mucR mutant

Genetic complementation of the *B. abortus mucR* mutant CC092^4^ with a wild-type version of the corresponding gene was performed as previously described^26^. The Q5 Site Directed Mutagenesis kit (NEB) was used to make *mucR*^*L36L39I40A*^ gene using the mutagenesis primers mucRL36AL39AI40A SDMF and R (Suppl. Table 1) and plasmid pJep011 which encodes the wild-type *Brucella mucR*^26^ as a template. The mutagenesis primers were designed using the web-based NEB Base Changer Program available at www.neb.com. The nucleotide sequence of the *mucR*^*L36L39I40A*^ gene confirmed by DNA sequence analysis.

The *B. abortus mucR* mutant CC092 was transformed by electroporation with either the pJep011^26^ or pJep120 plasmid and selected on Schaedlar agar supplemented with 5% defibrinated bovine blood (SBA)^4^ containing 45 μg/ml kanamcyin. The *B. abortus* strains were grown to mid log phase in brucella broth, and diluted in this medium to a cell density of approximately 10^4^ colony forming units (CFU) per ml. Twenty-five microliters (25 μ) of each bacterial cell suspension was spotted onto SBA plates, the plates incubated at 37 °C under 5% CO_2_, and the bacterial colonies produced by the individual strains observed and photographed after 72 h cultivation on this medium.

### q-RT PCR

Total RNA was isolated from *B*. *abortus* cells following growth to mid log phase in brucella broth (BD) using previously described procedures^4^. cDNA was generated from the final RNA preparation using the SuperScript III First Strand kit (Invitrogen) following the manufacturer’s protocol. The cDNA preparations were then used as the templates for real-time RT-PCR with SsoAdvanced Sybr Green Supermix (Bio-Rad) to evaluate the relative levels of gene-specific mRNA transcripts in the total cellular RNA preparations. The gene-specific oligonucleotide primers used for amplification of the experimental (*babR* [BAB1_0190], *bpdB* [BAB1_0512], *ftrA* [BAB2_0840], *btaE* [BAB1_0069], *mucR* [BAB1_0594] and control (*GAP* [BAB1_1741] and 16 S rRNA [BAB1_2223]) transcripts are listed in Supp. Table 1. The parameters used for the PCR reaction were a single denaturing step for 30 sec at 95 °C, followed by 40 cycles (denature for 15 sec at 95 °C, anneal/extend for 30 sec at 60 °C) of amplification. Fluorescence from SYBR green incorporation into double-stranded DNA was measured with a Bio-Rad CFX96 Thermocycler and analyzed on Bio-Rad Maestro Software. The results were confirmed by three biological and three technical replications. Data were analysed by the 2^−ΔΔCt^ method. Student T-test was used to evaluate the statistical significance of the results.

## Electronic supplementary material

Supplementary Information

## Data Availability

Data generated or analysed during this study are included in this published article (and its Supplementary Information files). The unpublished data are available from the corresponding author on reasonable request.
