# Supplementary material for: Identifying the region responsible for Brucella abortus MucR higher-order oligomer formation and examining its role in gene regulation
Source: Sci Rep. 2018 Nov 22;8:17238. doi: 10.1038/s41598-018-35432-1 (PMC6250670; doi:10.1038/s41598-018-35432-1)

Identifying the region responsible for *Brucella abortus* MucR higher-order oligomer formation and examining its role in gene regulation.

Luciano Pirone[2§], Joshua Edison Pitzer[3§], Gianluca D'Abrosca[1], Roberto Fattorusso[1], Gaetano Malgieri[1], Emilia Maria Pedone[2], Paolo Vincenzo Pedone[1*], Roy-Martin Roop II[3*], Ilaria Baglivo[1*].

[1] Department of Environmental, Biological and Pharmaceutical Sciences and Technologies, University of Campania, Caserta, 81100, Italy

[2] Institute of Biostructures and Bioimaging, C.N.R., Naples, 80134, Italy

[3] Department of Microbiology and Immunology, Brody School of Medicine, East Carolina University, Greenville, NC, USA

§ These two authors contributed equally as first authors of this article.

Supplementary Information.

Supplementary Table S1. Primer sequences for cloning and for q-RT-PCRs.

gene	Primer forward (5'- 3')	Primer reverse (5'- 3')
mucR$_{33-142}$*pet22b+*	CAGCATATGGCAGGCGAACTCCCGGTTCTG	CGGAATTCTCAGGCGTCCTTCGGCTTG
mucR$_{45-142}$-*pet22b+*	CAGCATATGGCTGCTTTCAAGCGCCAC	CGGAATTCTCAGGCGTCCTTCGGCTTG
mucR$_{33-142}^{L36L39I40A}$-*pet22b+*	CAGCATATGGCAGGCGAAGCCCCGGTTGCGGCTGCTGAAGTTCATGCTG	CGGAATTCTCAGGCGTCCTTCGGCTTG
mucR$_{L36L39I40A}$ *pet11Db+*	(mut primer): GGCGAAGCCCCGGTTGCGGCTGCTGAAGTTCATGCTGC (wild-type primer) CGGAATTCCATAATGGAAAATCTGGAAACG	(wild-type primer): CGGAATTCTCAGGCGTCCTTCGGCTTG (mut primer): CAGCAGCCGCAACCGGGGCTTCGCCTGCACGAATGG
mucR$_{57-142}$-*pet22b+*	CAGCATATGCCGGTCGTTGTTGAAAAGCCG	CGGAATTCTCAGGCGTCCTTCGGCTTG
mucR qPCR F2	ACCTGGTGACCCATTACAAC	GTTCGGAGCAACCATAGGATAG
16S qPCR	TCTCACGACACGAGCTGACG	CGCAGAACCTTACCAGCCCT
GAP qPCR	GCAGTTCGCGTCGCAAT	GAAGATGTGCGTTGGTTTCG
ftrA qPCR	ATCTCCAGCCCATCGAAATG	AAACGACCAGATAAGGCACC
btaE qPCR	CAAGGATGGGATTCGGCTAC	GTCTGGTCGGTATAGGCTTTC
bpdB qPCR	CTATCACATCAAGGATATCGGGC	GGCATAGATATAGGTTCCGTCC
babR qPCR	CACCAATCTCCTGCATCTATCC	GCTGATGTGGAAATTGACCG
mucRL36AL39AI40A SDMF	TGCGGCTGCTGAAGTTCATGCTGCTTTC	ACCGGGGCTTCGCCTGCACGAATGGA

Supplementary Table S2. Bacterial strains and plasmids used in this study.

Strain / plasmid	Genotype or description[a]
Escherichia coli	
DH5α	F- ϕ80 d*lacZ*ΔM15 Δ(*lacZYT-argF*)U169 *deoR recA*1 *endA*1 *hsdR*17 *phoA supE*44 I- *gyrA*96 *relA*1
BL21	F- *ompT* $hsdS_B$ *gal dcm*
Brucella abortus	
2308	Virulent challenge strain
CC092	2308 containing an isogenic *mucR* deletion
JEP15	CC092 complimented with pJep011
JEP120	CC092 complimented with pJEP120
Plasmids	
pET-22b(+)	Expression vector with T7 promoter; Amp^R
pET-11D	Expression vector with T7 promoter; Amp^R
pMR10	Broad host range, low copy vector; Kan^R
pJep011	*mucR* gene including the mucR promoter region in pMR10
pJep120	$mucR^{L36L39I40A}$ gene including the mucR promoter region in pMR10

[a]KanR – kanamycin resistance; AmpR – ampicillin resistance.

Supplementary Fig. S1. MucR and homologous proteins amino acid sequence alignment obtained by ClustalW (http://www.genome.jp/tools/clustalw/). The amino acids in red constitute the conserved hydrophobic region analysed in this paper.

```
MucR_Smeliloti        --MTETSLGTSNELLVELTAEIVAAYVSNHVVPVAELPTLIADVHSALNNTTAPAPVVVP
Ros_Atumefaciens      --MTETAYGNAQDLLVELTADIVAAYVSNHVVPVTELPGLISDVHTALSGTSAPASVAVN
Ml4                   MPLRRKPLTDENINLIELTADIVSAYVSNNPVPVASLPDLIHSVNLSLSKVGRPAE--PE
Ml3                   --MK-ELSNIEDKTVIELTADIVSAYVGNNPLPASGLPDLIASVSASVRKLAGAVV--VE
Ml1                   --MT-EEADKNIDTLIELTADVVSAYVSNNPVPVGDLPALIGQVHAALKGTAGFVS-AAK
Ml5                   --MT-EETESKADNLIELTAHVVSAYVSNNPVPVGELPGLIGQIHIALKGTAGGAA-PEK
Ml2                   MDIV-ETPSRNNDALIELTADVVAAYVSNNPVPVGELPNLISDVHAALGRVGGTAEQP-P
MucR_Babortus         MENL-ETNDESTELLLSLTADVVAAYVGNNSIRAGELPVLIAEVHAAFKRHVEREEAPVV
                                    ::.***.:*:***.*: : .  ** ** .:  :.
MucR_Smeliloti        VEKPKPAVSVRKSVQDDQITCLECGGTFKSLKRHLMTHHNLSPEEYRDKWDLPADYPMVA
Ros_Atumefaciens      VEKQKPAVSVRKSVQDDHIVCLECGGSFKSLKRHLTTHHSMTPEEYREKWDLPVDYPMVA
Ml4                   NPVLTPAVNPKKSVFPDYIVSLEDGRKFKSMKRHLG-LLGMTPDEYRTKWDLPRDYPMVA
Ml3                   SPSLVPAVNPKKSVFPDYIICLEDGKKFKSLKRHLRTDYGLSPDDYRAKWGLPPDYPMVA
Ml1                   PEALEPAVPIRKSVTPDYIICLDDGKKFKSLKRHLSTHHGLTPDEYRAKWHLPADYPMVA
Ml5                   SEALKPAVPIRKSVTPDYIISLEDGKKFKSLKRHLATHYGLTPDEYRAKWELPADYPMVA
Ml2                   ADKQKPAVNPKRSVHDDYIVCLEDGKKFKSLKRHLMTHYDLTPDQYREKWNLDPSYPMVA
MucR_Babortus         VEKPKPAVNPKKSVHDDYIVCLEDGKKFKSLKRHLVTHYNMTPEQYREKWDLDPNYPMVA
                           ***  ::**  * * .*: * .***:****    .::*::** ** *  .*****
MucR_Smeliloti        PAYAEALAKEMGLGQRRKRRGK-------------
Ros_Atumefaciens      PAYAEARRSRSRLAKEMGLGQRRKANR--------------
Ml4                   PNYAATRSALAKASGLGRKAAPVKKAPA-KRKAKA---
Ml3                   PNYSATRSALAKSTGLGRKPAAAPAAVAKKGKAKA---
Ml1                   PNYAAARSALAKTMGLGRKPKEPEARTRKKAAA-----
Ml5                   PNYAAARSALAKTMGLGRKPKEPETPAPAKRARKKAAA
Ml2                   PNYAAARSQLAKKMGLGRKRKAR---------------
MucR_Babortus         PNYAAARSRLAKKMGLGRKPKDA---------------
                      * *: :** ***  ***::
```

Supplementary Fig. S2. EMSA of MucR (a) and MucR^{L36L39I40A} (b) with Site 1 and with NC oligonucleotides as a negative control.
Three different amounts of the two proteins were tested with Site 1 (0.4, 0.6, 0.8 μg) and the increasing amount of protein is indicated on the top of the lanes 6-8 of panel a and b.
The double-stranded oligonucleotide Site 1 was also loaded onto the gel without protein in lane 8 panel a and b.
The double-stranded oligonucleotide, used as negative control (NC), was tested in the presence (lane 1 of panel a and b) or in the absence (lane 2 of panel a and b) of 0.8 μg of protein. In lane 3 of panel a and b, 0.8 μg of protein were tested with Site 1 (repeating the experiment shown in lane 7 in these two panels).
In lane 4 panel a and b 100 bp DNA Opti-DNA marker (abm) was loaded so that the complexes formed by MucR or MucR^{L36L39I40A} can be compared for migration and molecular mass.
The arrow indicates the MucR/DNA complex with a slower migration and higher molecular mass which is not present in the EMSAs of MucR^{L36L39I40A}.
Panel c shows only the Opti-DNA marker (abm) with the lengths of fragments.

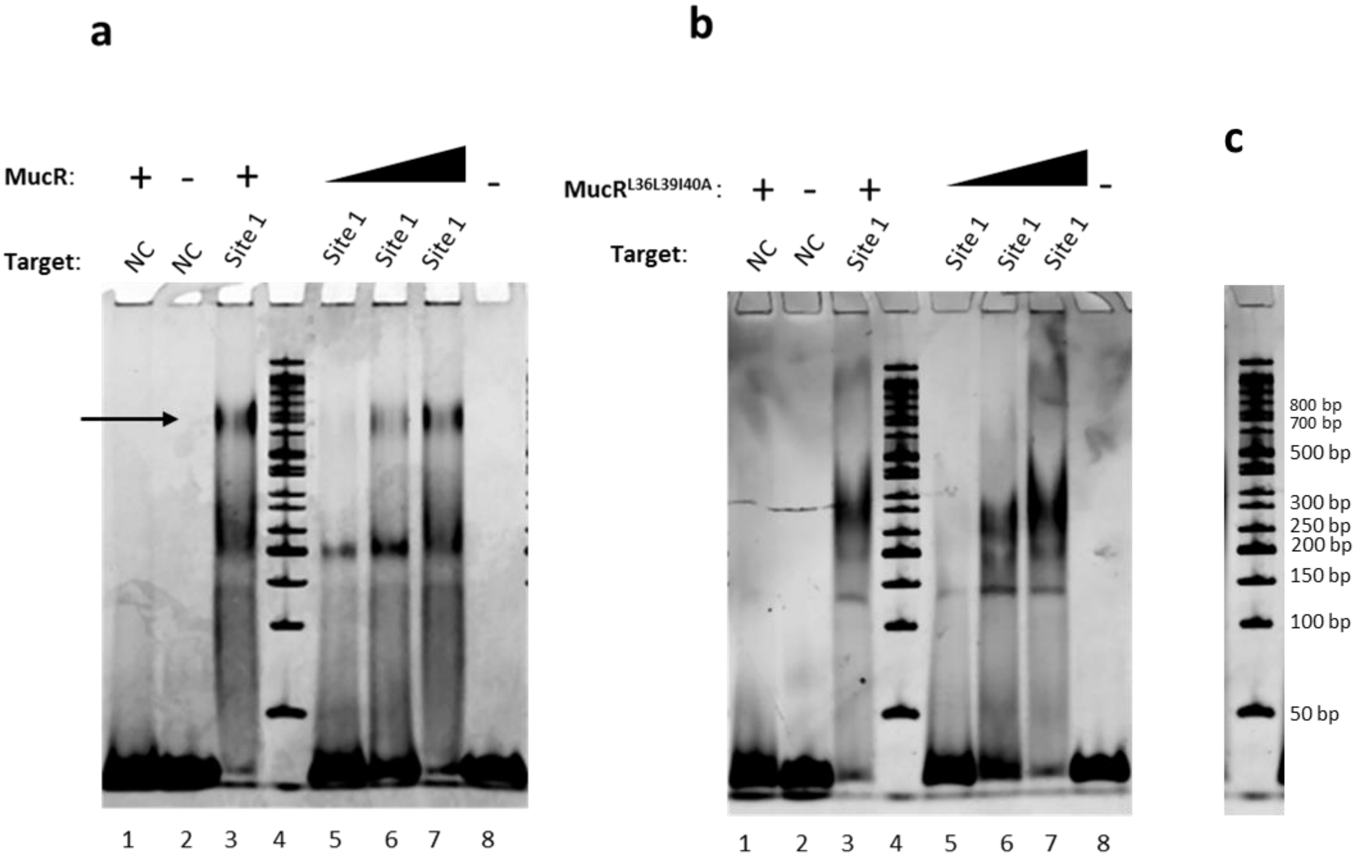

Supplement: Supplementary file 1 — Supplementary Information [file 41598_2018_35432_MOESM1_ESM.pdf]
